# Ecosystem carbon storage and carbon metabolizing microorganisms in three types of grasslands on the Qinghai-Tibet Plateau

**DOI:** 10.3389/fmicb.2025.1627840

**Published:** 2025-07-25

**Authors:** Qian Liu, Wenquan Yang, Jiancun Kou, Qinyao Li, Yangcan Zhang, Xilai Li, Jing Zhang, Zhiting Hao, Lu Chi, Yuze Ning

**Affiliations:** ^1^College of Grassland Agriculture, Northwest A&F University, Yangling, China; ^2^College of Life Sciences, Northwest A&F University, Yangling, China; ^3^College of Agriculture and Animal Husbandry, Qinghai University, Xining, China

**Keywords:** soil carbon functional microbial, carbon storage, alpine grassland, Tibetan Plateau, soil water content

## Abstract

**Background:**

The response of soil microorganisms to environmental changes can affect the storage and stability of carbon pools in ecosystems. However, the intrinsic link between the structure of soil carbon-metabolizing microbial communities and their roles in different types of alpine grasslands remains unclear.

**Methods:**

This study explores how carbon storage varies among alpine meadow (AM), alpine wetland (AW), and alpine desert (AD) on the Qinghai-Tibet Plateau and assesses the influence of a wide range of soil microbial and vegetation factors, so as to identify microbial predictors of ecosystem carbon storage. The study revealed four types of carbon metabolizing microbial communities responded to changes in vegetation types and their impact on the storage and stability of carbon pools.

**Results:**

The carbon storage of three grassland types followed the relativity of AW > AM > AD. Soil water content (SWC) was identified as the major factor affecting the carbon storage of grassland ecosystems by increasing vegetation belowground biomass and soil total carbon content, directly or indirectly influencing the diversity of four types of soil microorganisms through its effects on soil physicochemical properties. The community structure of these four types of carbon metabolizing microorganisms in AW significantly differed from that of AM and AD. The diversity of carbon-fixing microorganisms significantly reduced ecosystem carbon storage to a great extent. The relative abundance of carbon-fixing microorganisms *Thiobacillus*, *Mesorhizobium*, *Azospirillum*, and *Methylibium* significantly increased grassland carbon storage, while the relative abundance of chitinase-producing microorganisms *Cellulomonas* and *Stenotrophomonas* significantly decreased it.

## 1 Introduction

The Qinghai-Tibet Plateau, with an average altitude of over 4,000 m, is home to the world’s highest ecosystem, possessing an enormous potential for carbon sequestration and playing a crucial role in the global carbon balance (Chen H. et al., 2022; Canedoli et al., 2020). The alpine grassland on the Plateau covers more than 60% of its total area (Gilmanov et al., 2007). Although the soil is generally poor, the region is rich in vegetation and is one of the global biodiversity hotspots (Liu et al., 2018; Berdugo et al., 2019). Therefore, it is highly sensitive to changes in environmental conditions such as climate, vegetation, and soil physicochemical properties (Bai et al., 2012; Cleland et al., 2013).

Major grasslands atop the Qinghai-Tibet Plateau fall into three types of alpine meadow, alpine wetland, and alpine desert. Their distribution pattern is jointly shaped by water and heat. In the alpine environment of the Plateau, differential soil water content (SWC) is generally regarded as the key environmental factor driving the formation of different types of alpine grasslands (Du and Gao, 2020). Environmental changes (such as alterations in climate, vegetation, and soil properties) profoundly influence the carbon cycle processes in plateau grasslands (Ganjurjav et al., 2016; Yang et al., 2021). Among them, water availability (rather than mere precipitation) acts on carbon storage through multiple pathways (Du and Gao, 2020; Sun et al., 2022). It not only directly modulates plant growth (Deng et al., 2017; Sun et al., 2020) and vegetation carbon accumulation, but also, more critically, drives the soil carbon cycle by altering SWC, and thus affecting soil microbial communities (Li Y. et al., 2023; Li Z. et al., 2023). Soil microorganisms dominate the carbon transformation processes through catabolic and anabolic metabolism (Philippot et al., 2013; Liang et al., 2017). For instance, soil carbon-fixing microorganisms mediate the Calvin cycle (Ge et al., 2013), while methanogens and methanotrophs control methane production and oxidation, respectively (Juottonen et al., 2006), and chitinase-producing microorganisms are involved in the mineralization and decomposition of organic matter (such as chitin) (Brankatschk et al., 2011). Therefore, SWC regulates the core internal mechanisms of soil carbon pools (carbon fixation, transformation, and loss) in different grasslands by influencing the community structure, diversity, and activity of such key carbon-fixing microorganisms as methanogens, methanotrophs, and chitinase-producing microorganisms.

Previous studies have confirmed that soil microbial communities are susceptible to the nature of grassland and the associated environmental variables (such as vegetation characteristics and soil physicochemical properties) (Li Y. et al., 2023; Li Z. et al., 2023; Prober et al., 2015; Jones et al., 2018; Wang et al., 2024). Current research on microorganisms in alpine grasslands has focused primarily on the overall bacterial and fungal communities (Li Y. et al., 2023). Extremely scarce research has been carried out to specifically target functional microbial communities (carbon-fixing bacteria, methanogens, methanotrophs, and chitinase-producing bacteria) that drive core carbon cycling processes in different grasslands. As a result, it remains unknown how variations in SWC influence these key carbon-functional microbial communities and ultimately lead to differences in soil carbon pools among alpine meadows, wetlands, and deserts. Therefore, it is necessary to explore the community variation characteristics of carbon-metabolizing microorganisms in different types of grassland.

This study analyzes the differences in ecosystem carbon storage among three types of alpine grasslands, alpine meadow (AM), alpine wetland (AW), and alpine desert (AD) in the Qinghai-Tibet Plateau using the illumina sequencing technology. It focuses on the impact mechanisms of SWC on ecosystem carbon storage and soil carbon-metabolizing microbial communities in different grasslands. The objectives of this study are to: (1) investigate the differences in grassland carbon storage affected by SWC; (2) assess the influence of SWC on soil properties and vegetation characteristics among three types of alpine grasslands; (3) explore the influence of SWC on the diversity of carbon metabolizing microorganisms; and (4) ascertain the impact of soil carbon metabolizing microorganisms on grassland carbon storage. The results not only provide basic data for the study of carbon cycle and transformation in different types of alpine grasslands, but also provide reference and guidance for the study of carbon source and carbon storage control mechanism in different water conditions of alpine grassland.

## 2 Materials and methods

### 2.1 Study site

**Figure 1:**
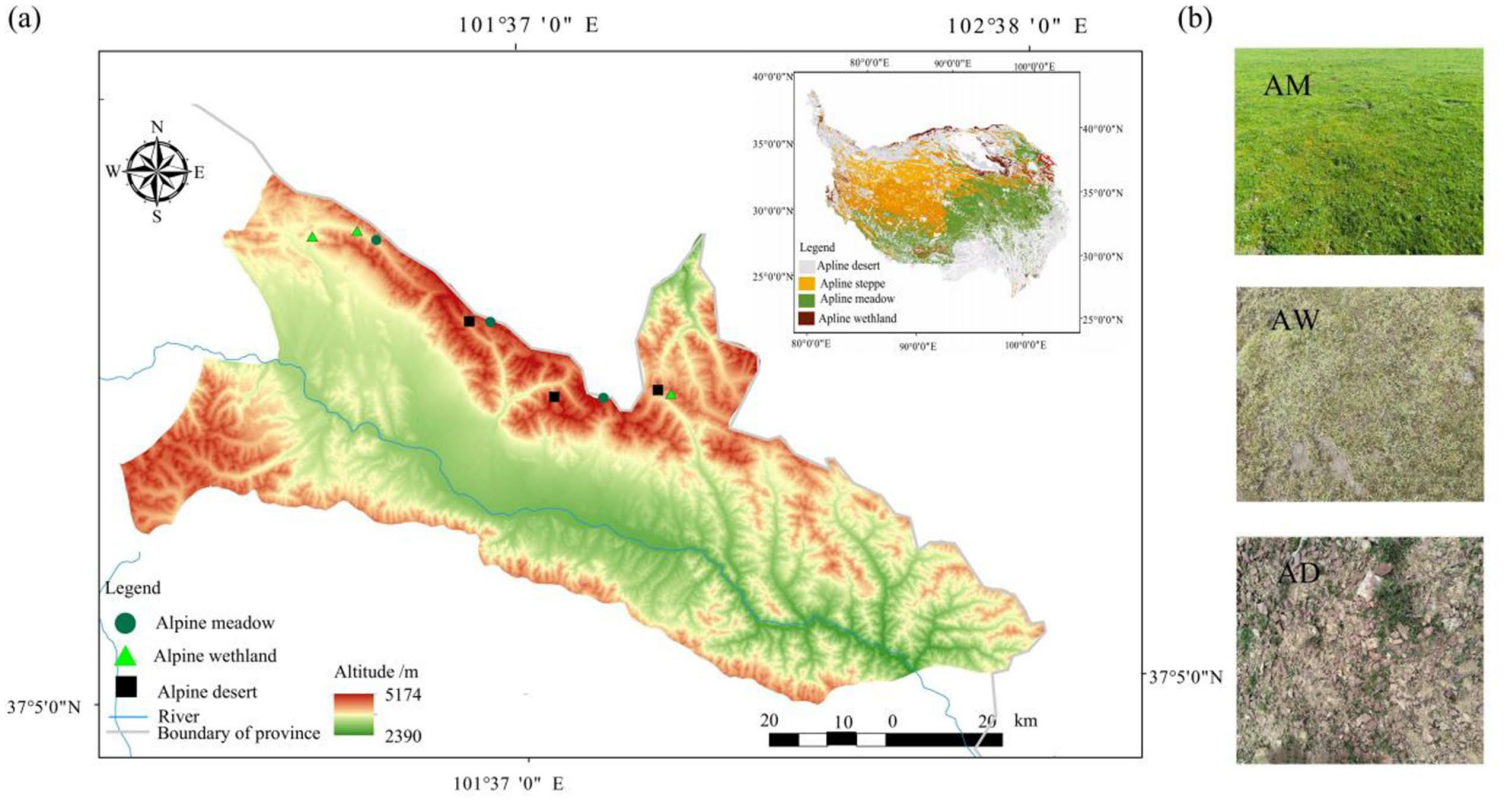
Sampling locations **(a)** and grassland types **(b)**. AM, alpine meadow; AW, alpine wetland; AD, alpine desert.

The experiments were conducted in Menyuan County, Haibei Prefecture, Qinghai Province, China (37°19′-37°47’N, 101°14′-102°1′E) (Figure 1a). The area has an average altitude of 3,681 m and a typical plateau alpine climate. The annual average temperature is 0.8°C, with a daily temperature range of 11.6–17.5°C, and the annual average precipitation is 520 mm. Alpine meadow is the main grassland type, and the soil is plateau meadow soil (Nachtergaele et al., 2012). The main dominant plants are *Kobresia myosuroides*, *Carex* spp., and *Elymus dahuricus*. Additionally, there are also pockets of alpine wetland and alpine desert. The former is mainly distributed in low-lying flat areas between mountains, with the dominant plants being *Carex* spp., *Kobresia myosuroides*, and *Polygonum aviculare*. The latter is primarily located near seasonal snow-covered zones on mountain tops, dominated by *Saussurea pulchra* and *Leontopodium leontopodioides*. The appearance of the three types of alpine grasslands is shown in Figure 1b. To account for regional heterogeneity, each type was replicated by selecting three spatially distributed sites at a distance of >5 km from each other. Each site-type combination was treated as an independent replicate to validate vegetation-specific differences in carbon storage and microbial communities.

### 2.2 Soil and plant sample collection

Soil and plant samples were collected from July 28 to July 31, 2023 on sunny days for the three typical types of alpine grasslands (AM, AW and AD) at three experimental sites each (Figure 1). Within each site, three random plots (10 m × 10 m) serving as three replicates were established, with a distance of >10 m between adjoining plots. Three quadrats (1 m × 1 m) were set up in each plot (Supplementary Figure S1). After vegetation survey in each quadrat, above-ground parts were collected and all above-ground parts of plants in the same quadrat were clipped to determine vegetation biomass by types of plants (grasses, sedges and forbs), the total biomass of the quadrat and subsequent carbon content analysis. The three-replicate average was regarded as the final results for each analyzed parameter. After clearing the surface cover, soils were sampled to a depth of 0–20 cm using a soil auger with a diameter of 5 cm. Five soil cores were taken from each plot, resulting in a total of 15 soil cores from the same plot (10 m × 10 m) (total sampled area = 15 × *π* × (0.05/2)^2^ ≈ 0.029 m^2^). They were then mixed to form one composite soil sample for the subsequent collection of vegetation roots. A total of 9 soil samples were collected from 3 kinds of grasslands for calculating the root biomass of vegetation (3 types of grassland × 3 sites). According to the above method, five soil cores were additionally collected from each plot and mixed for the analysis of microorganisms and soil physical and chemical properties. Immediately, a portion of it was transferred into 1.5 mL cryogenic vials, rapidly placed into a low-temperature storage container, and dispatched immediately to Shanghai Personalbio Biotechnology Co., Ltd. (Shanghai, China) for microbial analysis. In total, 27 soil samples were collected for the three types of grassland (3 types of grassland × 3 sites × 3 replicates).

### 2.3 Soil physicochemical properties and vegetation characteristics analysis

In the laboratory, the collected soil samples were treated by differential processing. The composite sample mixed with 15 soil cores was continuously rinsed with flowing water to separate plant roots from soil particles, and then dried in an oven at 65°C until the weight was stable. The dried roots and aboveground biomass were homogenized through 0.25 mm mesh grinding for plant carbon quantification using potassium dichromate oxidation (Li et al., 2017). The soil collected for the analysis of soil physical and chemical properties was divided into two parts. The first portion was air-dried and sieved through a 0.149 mm mesh to determine soil total carbon content and soil physicochemical properties, including soil total nitrogen (TN), ammonium nitrogen (NH_4_^+^-N) and nitrate nitrogen (NO_3_^−^-N) using the AA3 continuous flow analyzer (Auto Analyzer 3-AA3). Soil organic matter (SOM) was determined by the potassium dichromate-sulfuric acid oxidation method (Fu et al., 2004). Total phosphorus (TP) and available phosphorus (AP) of the soil were analyzed using ultraviolet spectrophotometer (UV-2450). Soil total carbon was measured with a soil total carbon analyzer (Primacs ATC 100 analyzer, Skalar, Netherlands). Soil total potassium (TK) was determined using an atomic absorption spectrometer (Shimadzu A6300, Japan). The second fraction was preserved for characterizing soil physical properties, including soil bulk density (BD), SWC, and pH. The first two were measured gravimetrically after 2 mm sieving (Wang et al., 2016), BD was determined by drying the sampled soil at 105°C to a constant weight. SWC was determined by oven-drying fresh samples in pre-weighed aluminum tins at 105°C to constant weight, with values calculated from mass loss. Soil pH was determined using a Palintest SKW500 sensor at a soil-water ratio of 1:2.5.

### 2.4 Carbon storage calculation

Soil carbon storage was calculated using the following formula (Kumar et al., 2012) (Equation 1):


(1) $$\text{Soil TCstock}=\sum_{i=1}^{n}(\mathrm{TC}\times \mathrm{BD}\times \mathrm{di})$$

TC: total carbon; BD: soil bulk density (g cm^−3^); di: soil layer thickness (cm) (its value was taken as the maximum of the 0–20 cm depth).

Plant carbon stock, including above-ground and below-ground parts of plants, was calculated according to the following formula (Li et al., 2017) (Equations 2, 3):


(2) Xstock=X×C

(3) PlantCstock=shootCstock+rootCstock

Where X represents the measured plant biomass (g m^−2^) (above-ground or root biomass), while C stands for the carbon content of above-ground parts and roots (g kg^−1^) of plants.

The formula for calculating ecosystem carbon stock was as follows (Wang et al., 2023) (Equation 4):


(4) EcosystemCstock=PlantCstock+SoilTCstock

### 2.5 Sequencing and raw data analysis of *pmoA*, *cbbL*, *mcrA*, and *chiA* genes

Soil genomic DNA was extracted with the OMEGA Soil DNA Kit (Omega Bio-Tek, United States). Microbial community profiling involved PCR amplification of target regions was carried out using primers listed in Table 1, with the thermal cycling parameters set as: initial denaturation at 95°C (3 min), 27 cycles of 95°C (30 s), 55°C (30 s), and 72°C (45 s), concluding with final extension at 72°C (10 min). Purified amplicons were sequenced on the Illumina MiSeq PE300 platform (Majorbio Bio-Pharm, China).

**Table 1:** Specific primers, fragment lengths and annealing temperature of functional genes.

Genes	Primer sequences (5′ − 3′)	Length (bp)	Annealing temperature	References
*pmoA*	A189F: GGNGACTGGGACTTCTGG	460	50	Bourne et al. (2001)
	V2r: GCCTTCSAGCTTGCCSACCRC			
*cbbL*	K2f: ACCAYCAAGCCSAAGCTSGG	492	62	Wang et al. (2021)
	V2r: GCCTTCSAGCTTGCCSACCRC			
*mcrA*	mcrA-F: GGTGGTGTMGGATTCACACARTAYGCWA	460	55	Zhou et al. (2014)
	mcrA-R: TTCATTGCRTAGTTWGGRTAGTT			
*chiA*	chif2: GACGGCATCGACATCGATTGG	500	55	Xiao et al. (2005)
	chir: CSGTCCAGCCGCGSCCRTA			

Bioinformatic processing included primer removal via cutadapt and sequence deduplication using the derep_fulllength module. Chimeric sequences were eliminated through uchime_denovo analysis. High-quality sequences were clustered into operational taxonomic units (OTUs) at the 97% similarity using the cluster_size module. Sequence normalization was achieved using rarefaction analysis (Kemp and Aller, 2004) to standardize sequencing depth across samples, enabling comparative assessment of OTU distributions and relative abundances. Representative sequences and OTU tables were generated for subsequent ecological analyzes.

### 2.6 Statistical analysis

SPSS 26.0 software (IBM Corporation, Armonk, NY, United States) was utilized to conduct a one-way analysis of variance (ANOVA), followed by a Tukey test to evaluate the characteristics of different types of alpine grassland plants, soil physicochemical properties, *α*-diversity of soil carbon metabolizing microorganisms, and the relative abundance of soil carbon metabolizing microbial phyla and genera. Origin 2018 was used to create bar charts of carbon components across different grassland types. Based on the OTU analysis results, the vegan package in R (v.3.5.2) was employed to calculate α diversity indices (Chao1 and Shannon), perform principal coordinate analysis (PCoA), and construct random forest models for species. The vegan and ggplot2 modules in R (v.3.5.2) were utilized to create redundancy analysis (RDA) plots of environmental factors and carbon-metabolizing microbial communities. Differences in the composition of carbon-metabolizing microorganisms among different types of alpine grasslands were tested using a matrix based on Bray–Curtis dissimilarity, and visualized through non-metric multidimensional scaling (NMDS) and PCoA based on Bray–Curtis dissimilarity. Non-parametric tests consistent with multivariate analysis of variance (ADONIS, ANOSIM, MRPP) were used to assess the significance of these differences. Venn analysis was conducted to determine the number of shared and unique OTUs among the three types of alpine grasslands. Potential microbial biomarkers were obtained using the Linear Discriminant Analysis Effect Size (LEfSe) method. Correlation analysis and Mantel tests were conducted to investigate the relationships between carbon storage, vegetation characteristics, soil physicochemical properties, and *β*-diversity and species richness of carbon-metabolizing microbial communities. Random forest modeling was used to predict the relative importance of *α* and *β* diversity indices of soil carbon-metabolizing microorganisms, and the relative abundance of key genera of carbon metabolizing microorganisms and their impact on carbon storage. Predictors with negative IncMSE values were excluded prior to model fitting. The final random forest model, optimized at 1000 trees via cross-validation, quantified variable importance through permutation-based significance testing.

Partial Least Squares Structural Equation Modeling (PLS-SEM) was carried out using SmartPLS 4.0 to quantify the direct and indirect effects of SWC on carbon storage and carbon metabolizing microorganisms across the grassland types. PLS-SEM was selected for its robustness with a limited sample size and abnormally distributed data (Jöreskog and Goldberger, 1975; Hair et al., 2019). Within this framework, we analyzed the influence of SWC on soil physical and chemical properties (pH, BD, TC, TN, TP, TK, SOM, NO_3_^−^-N, NH_4_^+^-N and AP), vegetation characteristics (AGB and BGB), and the diversity of carbon-metabolizing microorganisms (Shannon and Simpson indices), as well as the pathways and extent of their impact on the carbon storage of grassland ecosystems. Model validity was assessed using three metrics: the coefficient of determination (*R*^2^), predictive relevance (Q^2^), and goodness of fit (GoF). An *R*^2^ value of approximately 0.67 is considered substantial, a value around 0.33 indicates a moderate explanatory power, and a value around 0.19 suggests a weak explanatory power. When Q^2^ is greater than 0, the SEM model has a higher predictive relevance. A GoF value less than 0.1 indicates a weak explanatory power, around 0.25 a moderate explanatory power, and around 0.36 a strong explanatory power (Lu et al., 2024). The predictive performance of PLS-SEM was compared with that of the data-driven random forest method to illustrate the applicability of PLS-SEM for predictive purposes.

## 3 Results

### 3.1 Characteristics of plants and soil in different types of alpine grassland

There were significant differences in plant characteristics among the three types of alpine grassland (*p* < 0.05). The aboveground biomass of grasses, sedges and forbs in AM was significantly higher than in the other two types of alpine grassland, and the Shannon-Wiener index of biomass and vegetation on miscellaneous grassland in AW was the lowest (*p* < 0.05). In contrast, vegetation coverage and belowground biomass were significantly higher in AW than in AM and AD (*p* < 0.05). Soil pH ranged from 6.52 to 7.13, and the soil pH of AD was significantly higher than that of AM and AW (*p* < 0.05). The contents of SWC, TC, TN, SOM and NH_4_^+^-N in AW were significantly higher than those in AM and AD, while the contents of BD and TK in AW were significantly lower than those in AM and AD (*p* < 0.05). Except for soil pH, there was no significant difference in the content of other soil physical and chemical properties between AM and AD. There was no significant difference in the contents of TP and NO_3_^−^-N among the three types of alpine grassland (Table 2).

**Table 2:** Plant characteristics and soil physicochemical properties of three types of alpine grassland.

Parameters	Grassland types		
	AM	AW	AD
Plant community indices			
AGB (g m^−2^)	388.56 ± 11.73a	178.78 ± 10.53b	96.33 ± 2.18c
BGB (g m^−2^)	3597.60 ± 219.59b	6419.31 ± 179.56a	1799.88 ± 592.49c
Grass−biom (g m^−2^)	64.76 ± 1.95a	25.24 ± 1.50b	13.76 ± 0.31c
Sedge−biom (g m^−2^)	259.04 ± 7.82a	128.82 ± 5.36b	48.17 ± 1.09c
Forb−biom (g m^−2^)	194.28 ± 5.86a	24.41 ± 8.80c	34.40 ± 0.78b
Coverage	65.07 ± 3.87b	75.11 ± 12.24a	26.56 ± 4.16c
Shannon	1.93 ± 0.28a	1.15 ± 0.42b	1.52 ± 0.35ab
Simpson	0.82 ± 0.06a	0.64 ± 0.12b	0.74 ± 0.11a
Soil indices			
pH	6.55 ± 0.13b	6.52 ± 0.46b	7.13 ± 0.21a
BD (g cm^-3^)	113 ± 11.98a	63.91 ± 27.01b	133.50 ± 15.81a
SWC (%)	21.02 ± 2.05b	49.38 ± 12.77a	11.95 ± 3.62b
TC (g kg^−1^)	34.05 ± 9.52b	93.53 ± 55.45a	12.18 ± 6.06b
TN (g kg^−1^)	3.26 ± 0.78b	7.87 ± 4.68a	1.46 ± 0.57b
TP (g kg^−1^)	0.79 ± 0.06a	0.73 ± 0.12a	0.83 ± 0.16a
TK (g kg^−1^)	23.88 ± 1.16a	21.53 ± 1.73b	24.77 ± 0.47a
SOM (g kg^−1^)	57.87 ± 16.71b	158.52 ± 93.57a	21.24 ± 9.79b
NO_3_^−^-N (mg kg^−1^)	26.53 ± 11.45a	30.06 ± 24.83a	14.46 ± 1.46a
NH_4_^+^-N (mg kg^−1^)	10.77 ± 3.53b	19.24 ± 3.92a	7.84 ± 1.11b
AP (mg kg^−1^)	6.57 ± 0.87ab	9.2 ± 4.60a	5.31 ± 0.58b

AGB, aboveground plant biomass; BGB, belowground biomass; Grass−biom, grass aboveground biomass; Sedge−biom, sedge aboveground biomass; Forb−biom, forb aboveground biomass; BD, bulk density; SWC, soil water content; TC, total carbon; TN, total nitrogen; TP, total phosphorus; TK, total potassium; SOM, soil organic matter; NO_3_^−^-N, nitrate nitrogen; NH_4_^+^-N, ammonium nitrogen; AP, available phosphorus; a, b, and c indicate significant differences at the 0.05 level.

### 3.2 Carbon component differences across alpine grassland types

**Figure 2:**
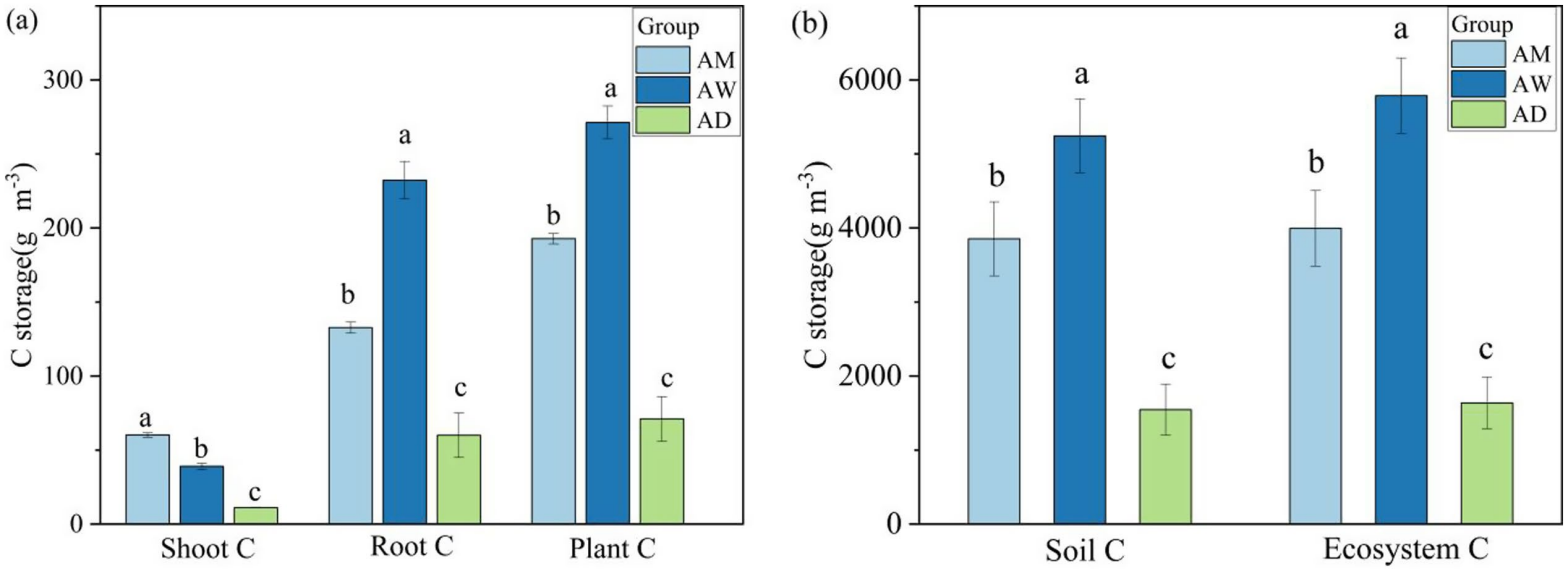
Differences in carbon storage in plant **(a)** and in soil and ecosystems **(b)** among three types of alpine grassland. AM, alpine meadow; AW, alpine wetland; AD, alpine desert. Different letters indicate significant differences at the 0.05 level.

Among the three types of alpine grassland, the highest carbon storage was found in the soil, accounting for over 90% of the ecosystem’s total carbon, followed by plant roots, with the lowest carbon storage in the belowground parts of plants (Figure 2a). Significant differences in carbon components were observed among AM, AW, and AD (*p* < 0.05). Each carbon component was lower in AD than in AM and AW (*p* < 0.05). The carbon storage in plant roots, vegetation, soil, and the entire ecosystem was the highest in AW (*p* < 0.05). However, the belowground vegetation carbon storage was significantly higher in AM than in AW and AD (*p* < 0.05) (Figure 2b).

### 3.3 Microbial community diversity and community composition of soil carbon metabolism

**Figure 3:**
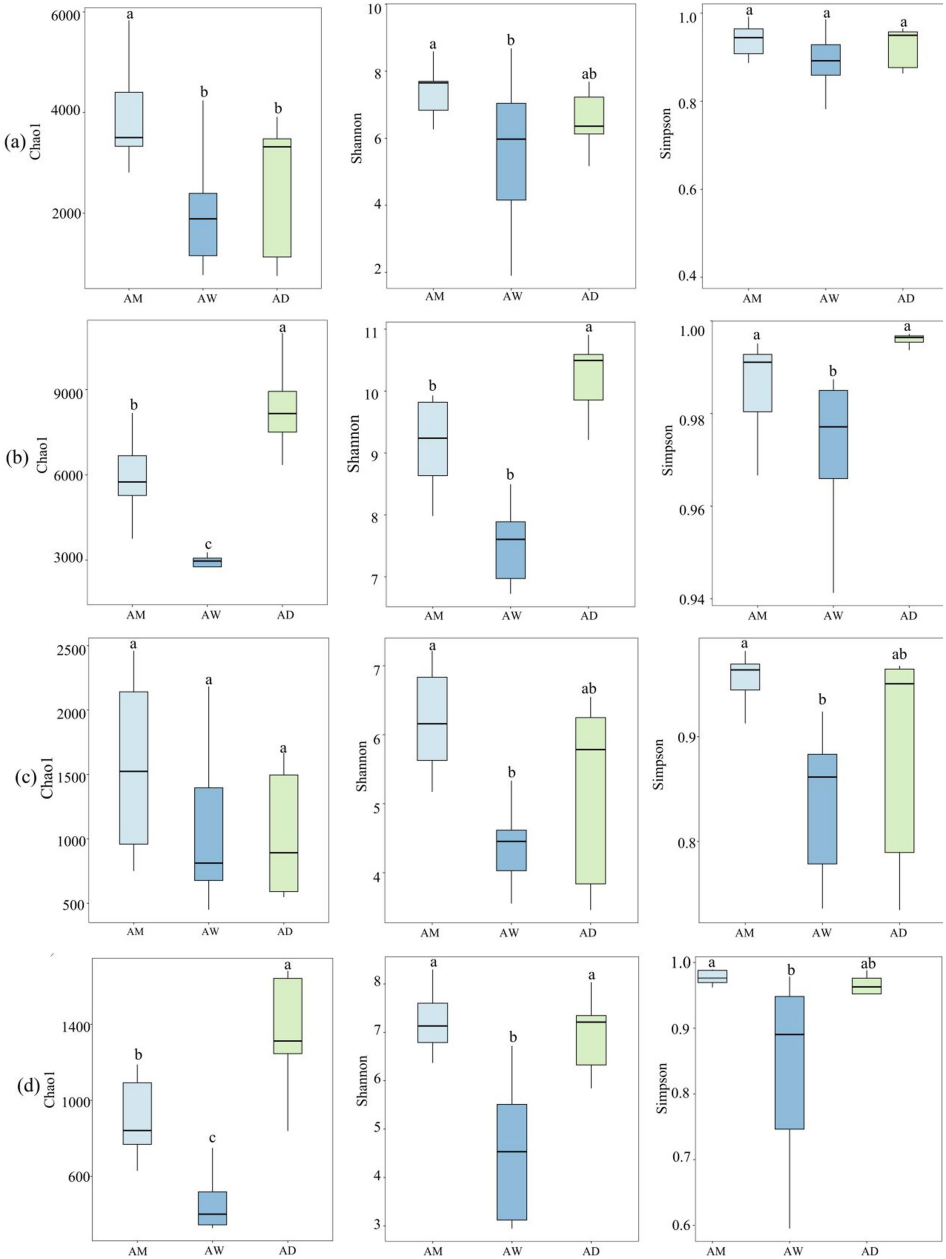
Box plots of *α* diversity index of soil carbon metabolizing microorganisms in three types of alpine grassland. They show Chao1, Shannon and Simpson indices of methanotrophs **(a)**, carbon-fixing microorganism **(b)**, methanogens **(c)**, and chitinase-producing microorganism **(d)** communities. AM, alpine meadow; AW, alpine wetland; AD, alpine desert. Different letters indicate significant differences at the 0.05 level.

The *α* diversity indices of soil microbial communities encompass richness indices and diversity indices, characterized by the Chao1, Shannon, and Simpson indices, respectively. Results from the Spearman analysis, as shown in Figure 3, indicate differences in the diversity and richness of soil carbon-metabolizing microbial communities among the three types of alpine grasslands. The Chao1, Shannon, and Simpson indices of the four types of carbon-metabolizing microorganisms were the lowest in AW, while the Chao1 index and Shannon index of methanotrophs were significantly higher in AM than in AW (*p* < 0.05) (Figure 3a). The trends in the Chao1 index, Shannon index, and Simpson diversity of soil carbon-fixing microorganisms were consistent across the three types of alpine grasslands, with the lowest values found in AW and the highest in AD (Figure 3b). The Chao1 value of the methanogens community showed little variation, whereas the Shannon and Simpson indices were significantly higher in AM than in AW (*p* < 0.05) (Figure 3c). The Chao1 value of the chitinase-producing microorganism community was significantly higher in AD than in AM, while no significant difference in the Shannon and Simpson indices existed between AM and AD (Figure 3d).

**Figure 4:**
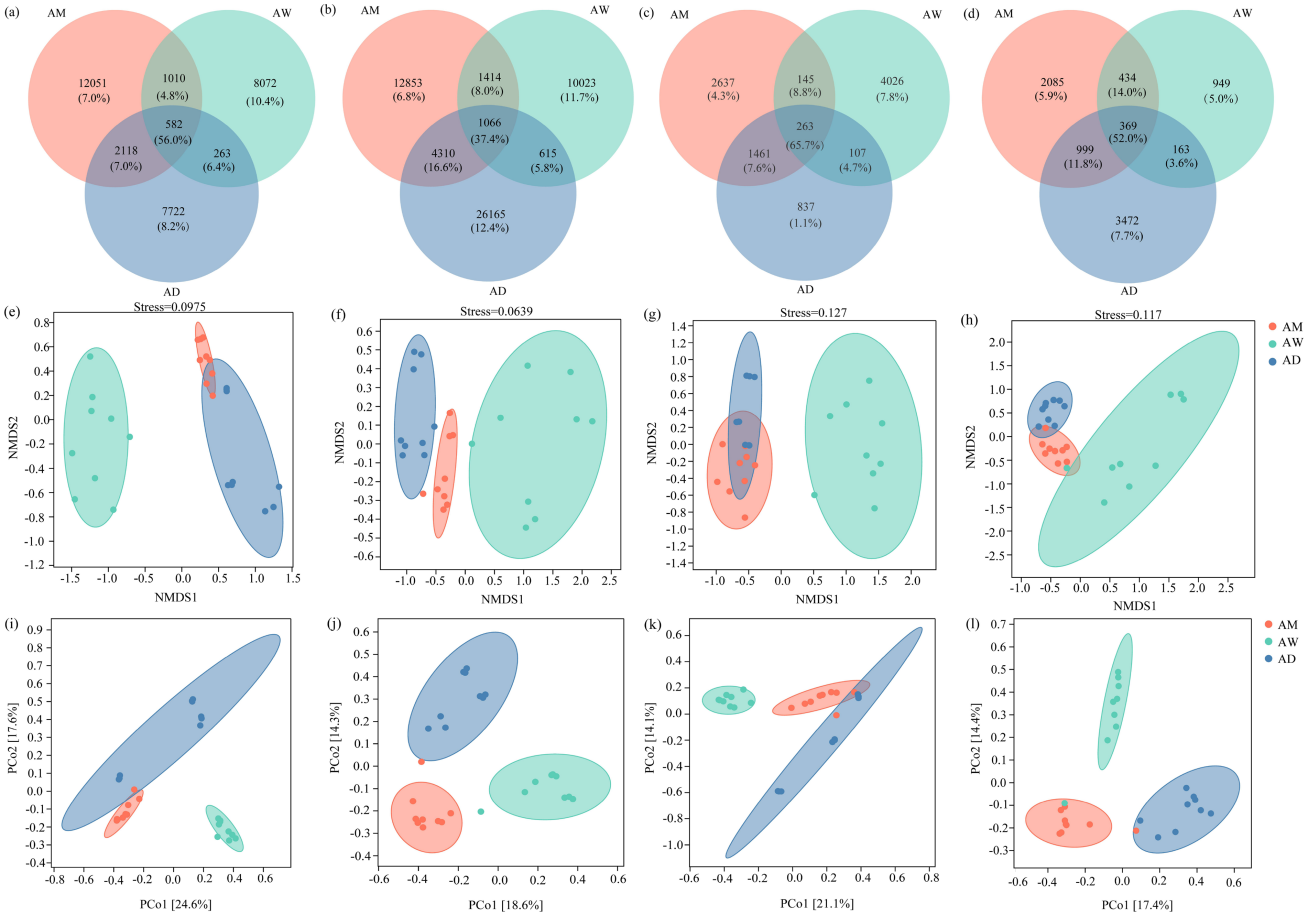
Soil carbon metabolizing microbial *β* diversity in three types of alpine grassland. The Venn diagram shows the number of unique and common OTUs of methanotrophs **(a)**, carbon-fixing microorganism **(d)**, methanogens **(g)**, and chitinase-producing **(j)** microorganisms communities; NMDS analysis of methanotrophs **(b)**, carbon-fixing microorganism **(e)**, methanogenic **(h)** and chitinase-producing microorganisms **(k)**; Principal coordinate analysis (PCoA) of OTU level methanotrophs **(c)**, carbon-fixing microorganism **(f)**, methanogens **(i)**, and chitinase-producing microorganisms **(l)** communities based on Bray-Curtis distances. AM, alpine meadow; AW, alpine wetland; AD, alpine desert.

The Venn diagram showed that the number of unique OTUs for methanotrophs and methanogenic microorganisms was the highest in AW (Figures 4a,c), while the number of unique OTUs for carbon-fixing microorganisms and chitinase-producing microorganisms was the highest in AD (Figures 4b,d). The three types of alpine grasslands shared 582 (56.0%) OTUs for methanotrophs, 1,066 (37.4%) OTUs for carbon-fixing microorganisms, 263 (65.7%) OTUs for methanogens, and 369 (52.0%) OTUs for chitinase-producing microorganisms (Figures 4a–d).

NMDS and PCoA results revealed discernible differences in the composition of the soil carbon-metabolizing microbial community among the alpine grasslands. Specifically, the communities of methanotrophs, carbon-fixing microorganisms, methanogens and chitinase-producing microorganisms in AW were significantly more distinctive from each other than they were in AM and AD (Figures 4e–h), indicating significant differences in the structure of the soil carbon metabolizing microbial community between AW and AM/AD. The carbon-fixing microorganism communities were significantly separated among AM, AW, and AD (Figure 4f), suggesting significant differences in the soil carbon-fixing microbial community structure among the alpine grasslands. PCoA revealed that, at the OTU level, there were significant differences (*p* < 0.05) in the soil carbon metabolizing microbial communities among different types of alpine grasslands, with the first two principal coordinate axes explaining 42.2, 32.9, 35.2, and 31.8% of the total variation for methanotrophs, carbon-fixing microorganisms, methanogens, and chitinase-producing microorganisms, respectively (Figures 4c,f,i,l).

**Figure 5:**
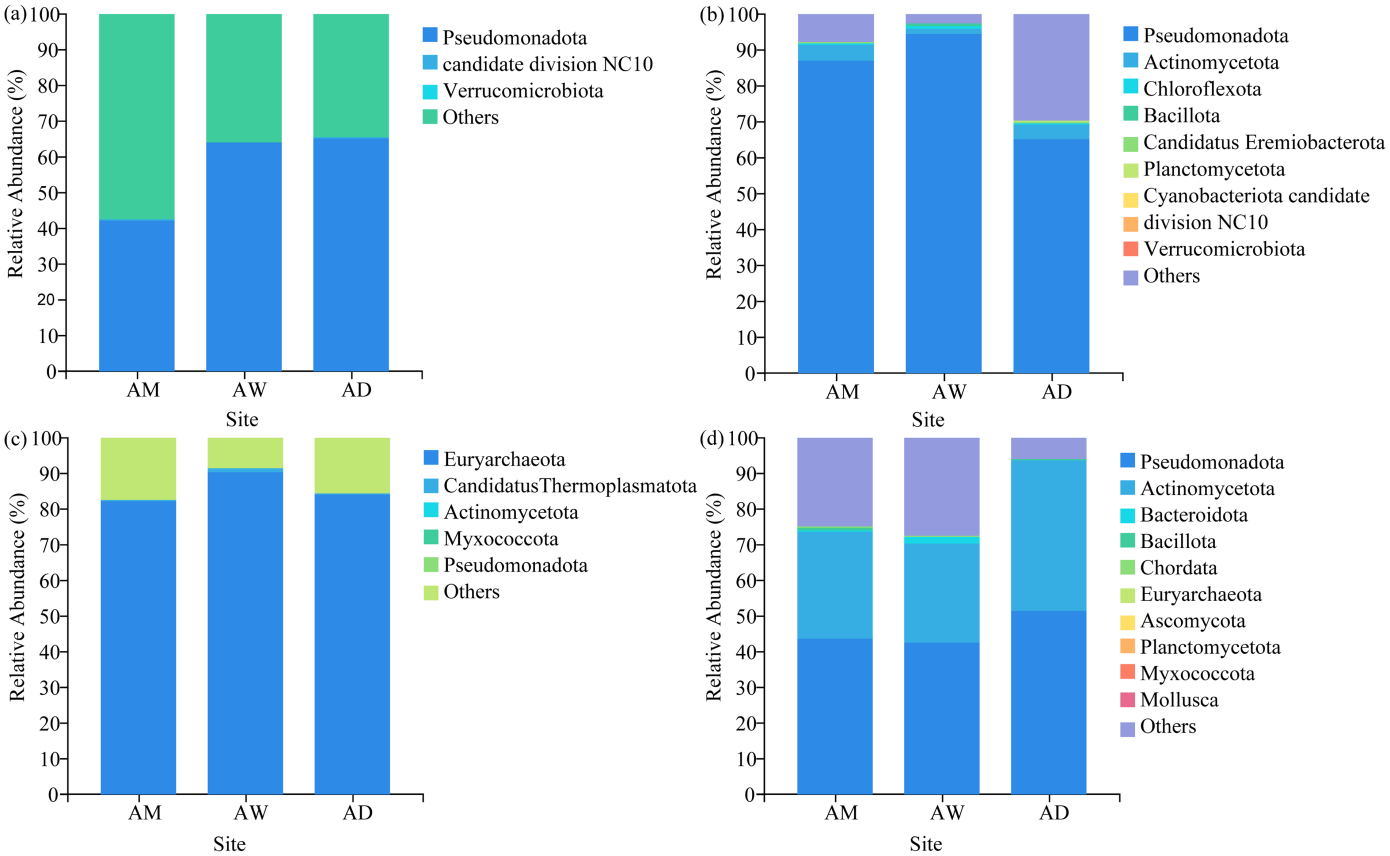
Soil community composition of **(a)** methanotrophs, **(b)** carbon-fixing microorganism, **(c)** methanogens and **(d)** chitinase-producing microorganism at the phylum level. AM, alpine meadow; AW, alpine wetland; AD, alpine desert.

**Figure 6:**
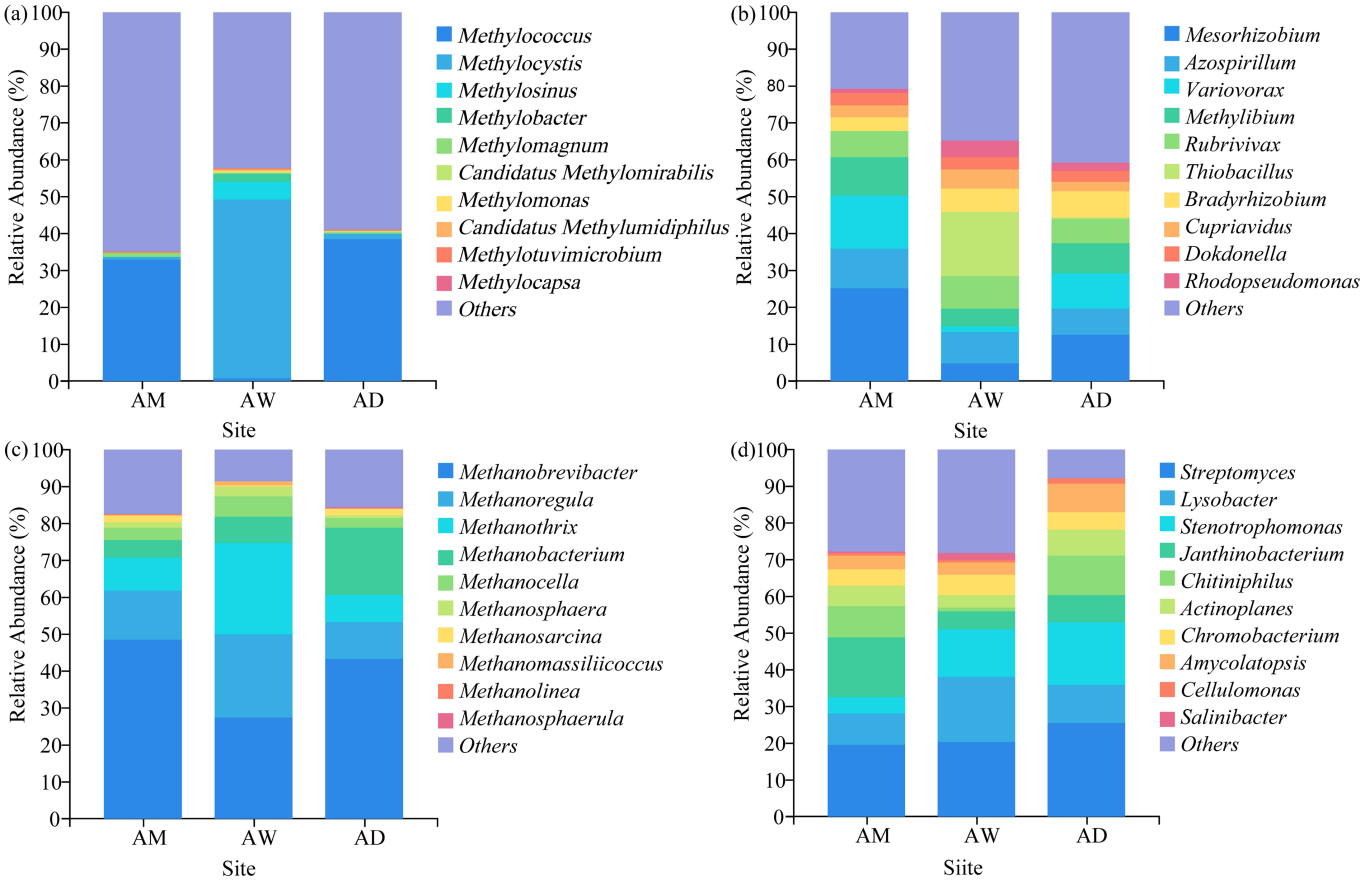
Soil community composition of **(a)** methanotrophs, **(b)** carbon-fixing microorganism, **(c)** methanogens and **(d)** chitinase-producing microorganism at the genus level. AM, alpine meadow; AW, alpine wetland; AD, alpine desert.

In terms of classification and composition, at the phylum level, the dominant phylum of all types of methanotrophs and carbon-fixing microorganisms in AM was Pseudomonadota, and the dominant phylum of methanogens was Euryarchaeota. The dominant phyla of chitinase-producing microorganisms were Pseudomonadota and Actinomycetota (Figure 5). At the genus level, the three types of alpine grasslands differed in their taxonomic composition of methanotrophs. Specifically, AM and AD were dominated by the *Methylococcus* (33.08–38.56%) species in the methanotrophs community, AW was dominated by the *Methylocystis* (10.25–92.84%) genus (Figure 6a). The top-10 genera components (in relative abundance) of carbon-fixing microorganism community were identified as *Mesorhizobium* (4.64–25.08%), *Azospirillum* (7.30–10.67%), Var*iovorax* (1.41–14.69%), *Methylibium* (4.85–10.37%), *Rubrivivax* (6.61–9.21%), *Thiobacillus* (0.05–17.34%), *Bradyrhizobium* (3.67–6.14%), *Cupriavidus* (2.43–5.31%), *Dokdonella* (3.06–3.62%), and *Rhodopseudomonas* (1.07–4.61%) (Figure 6b). Among them, the relative abundance of *Mesorhizobium*, *Azospirillum*, and *Methylibium* was the highest in AM and the lowest in AW. In contrast, the relative abundance of *Thiobacillus* and *Rhodopseudomonas* was significantly higher in AW than in AM and AD (*p* < 0.05). The dominant components of methanogens community were *Methanobrevibacter* (27.52–48.57%) and *Methanoregula* (10.06–22.46%), and there were no significant differences in the composition of methanogens community among the three types of alpine grasslands (Figure 6c). In the composition of chitinase-producing microorganism community, the genera with an average relative abundance greater than 1% were *Streptomyces* (19.49–25.56%), *Lysobacter* (8.57–17.68%), *Stenotrophomonas* (4.34–17.03%), and *Janthinobacterium* (5.04–16.42%). Among them, the relative abundance of *Streptomyces* and *Stenotrophomonas* in AD was significantly higher than that in AM (*p* < 0.05) (Figure 6d).

LEfSe analysis revealed that there are 65 taxonomic groups of soil carbon metabolism microorganisms, including 2 kingdoms, 4 phyla, 10 classes, 13 orders, 12 families, and 14 genera. They showed statistically significant differences among the three types of grassland. Specifically, *Methylosinus* and *Methylacidiphilum* are the dominant methanotrophs in AW (Supplementary Figure S2a). *Mesorhizobium*, Var*iovorax*, and *Methylibium* dominate the carbon-fixing microorganisms in AM, while *Thiobacillus*, *Rhodopseudomonas*, and *Sulfuricaulis* exhibit an absolute dominance in AW (Supplementary Figure S2b). *Methanobrevibacter* and *Methanosphaera* show a clear dominance in AW and AD, respectively (Supplementary Figure S2c). *Stenotrophomonas*, *Chitiniphilus*, and *Amycolatopsis* are the dominant chitinase-producing microorganisms in AD, while Janthinobacterium exhibit a clear dominance in AM (Supplementary Figure S2d).

### 3.4 Relationships between soil carbon-metabolizing microbial communities and environmental factors

**Figure 7:**
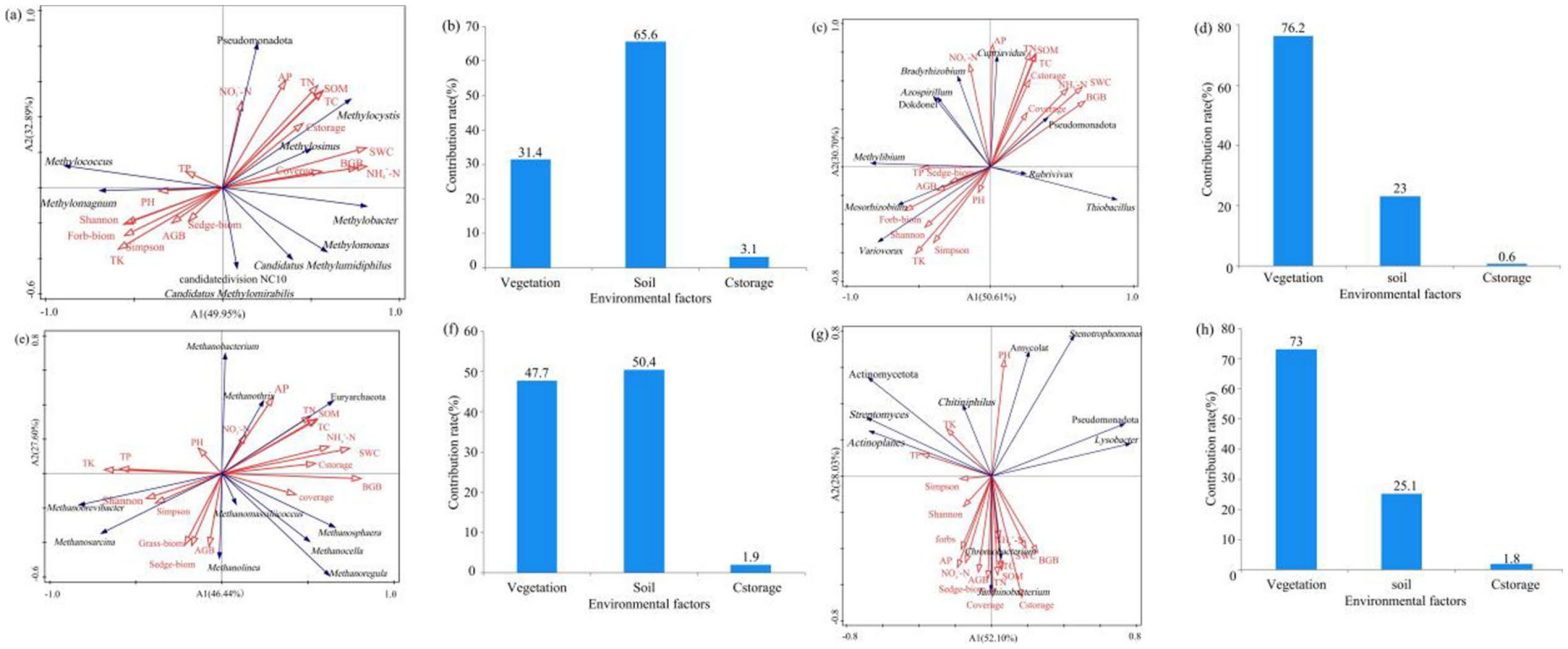
RDA results showing the relationship between environmental factors and soil **(a)** methanotrophs, **(c)** carbon-fixing microbial, **(e)** methanogens, and **(g)** chitinase-producing microbial communities; contribution of influencing factors to the diversity of **(b)** methanotrophs, **(d)** carbon-fixing microbial, **(f)** methanogens, and **(h)** chitinase-producing microbial communities. The contribution rate of soil physicochemical properties is the sum of the contribution rates of pH, BD, SWC, TC, TN, T, TK, SOM, NO_3_^−^-N, NH_4_^+^-N, and AP. The contribution rate of vegetation is the sum of the contribution rates of ABG, BGB, Sedge−biom, Grass−biom, Forb−biom, Shannon, Simpson, and coverage.

Based on the Mantel test results, soil characteristics (SOM, NO_3_^−^-N, NH_4_^+^-N, AP, TN, TC, SWC) and BGB were found to be significantly positively correlated with carbon storage (*p* < 0.01) (Figure 7a). Carbon storage was significantly correlated with the community structure of methanotrophs and carbon-fixing microorganisms. The community structure of methanotrophs was significantly correlated with NH_4_^+^-N (*r* = 0.424, *p* = 0.001; *r* = 0.495, *p* = 0.001) (Supplementary Table S1). Except for the methanogens community, soil physicochemical properties and vegetation characteristics were significantly correlated with the community structure of methanotrophs, carbon-fixing microorganisms, and chitinase-producing microorganisms. Figure 7b showed that carbon storage was significantly negatively correlated with the diversity indices (Chao1, Shannon, and Simpson) of carbon-fixing microorganisms and the diversity indices (Chao1 and Shannon) of chitinase-producing microorganisms (*p* < 0.05).

RDA analysis of environmental factors and dominant phyla and genera of methanotrophs revealed a strong positive correlation between NO_3_^−^-N and Pseudomonadota, a strong positive correlation between pH and *Methylomagnum*, a strong positive correlation between carbon storage and *Methylocystis*, and a positive correlation between TP and *Methylococcus*, but a strong negative correlation between TP and *Methylomonas* (Figure 7a). RDA analysis of environmental factors and dominant phyla and genera of carbon-fixing microorganisms revealed a strong positive correlation between SWC and the dominant phylum Pseudomonadota of carbon-fixing microorganisms, a strong positive correlation between AP and *Cupriavidus*, and a strong positive correlation between TP and *Methylibium*. Additionally, sedge biomass, grass biomass, and total belowground biomass of vegetation were strongly positively correlated with *Mesorhizobium* but strongly negatively correlated with Pseudomonadota (Figure 7c). Soil TN, TC, and SOM were strongly positively correlated with the dominant phylum Euryarchaeota of methanogens, while vegetation Shannon and Simpson diversity indices were strongly positively correlated with the dominant genera *Methanobrevibacter* and *Methanosarcina* of methanogens, respectively, but strongly negatively correlated with the dominant phylum Euryarchaeota of methanogens (Figure 7e). Among plant characteristics, aboveground biomass and vegetation coverage were the main factors influencing the dominant genera *Janthinobacterium* and *Chromobacterium* of chitinase-producing microorganisms, and the vegetation Shannon and Simpson indices were strongly negatively correlated with the dominant phylum Pseudomonadota (Figure 7g). Furthermore, among soil physicochemical properties, TK was strongly positively correlated with the dominant phylum Actinomycetota of chitinase-producing microorganisms, TP was strongly positively correlated with the dominant genus *Actinoplanes*, and soil NH_4_^+^-N, TC, TN, and SOM were strongly positively correlated with the dominant genus *Chromobacterium* (Figure 7g).

Based on the RDA analysis results, the influential factors contributing to methanotrophs diversity were identified as soil physicochemical properties (a contribution of 65.6%), also the main factors affecting methanotrophs diversity (Figure 7b). Vegetation characteristics were the main factors influencing the diversity of carbon-fixing microorganisms, with a contribution rate of 65.6% (Figure 7d). The main factors influencing methanogens diversity were vegetation and soil characteristics, with a contribution rate of 47.7 and 50.4%, respectively (Figure 7f). Plant characteristics were the main factor affecting chitinase-producing microorganisms, with a contribution of 73.0% (Figure 7h).

**Figure 9:**
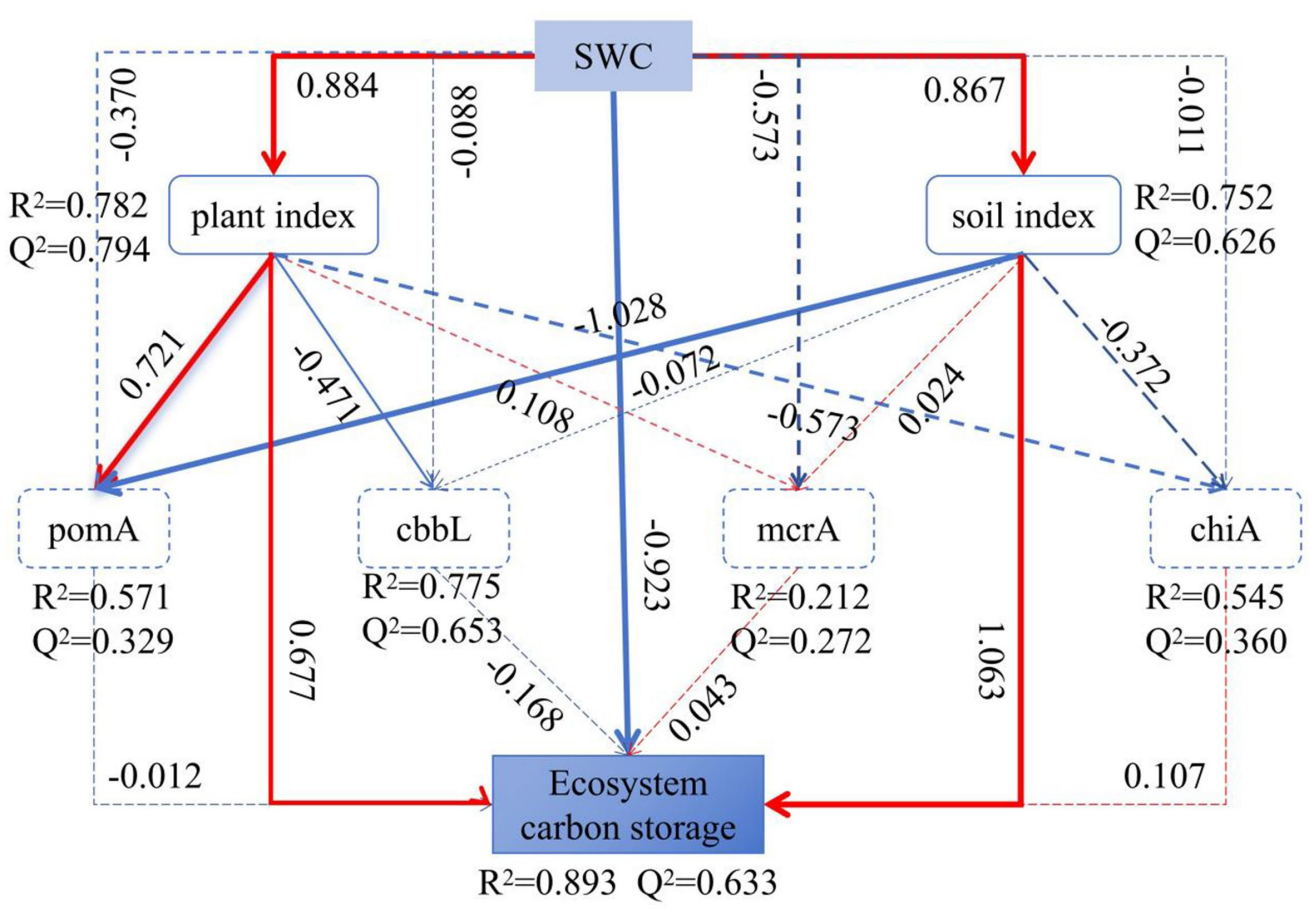
Partial Least Squares Path Modeling (PLS-SEM) of the impact of SWC on plants, soil characteristics, ecosystem carbon storage, and carbon metabolism microorganisms among three types of grassland. Solid and dashed lines indicate significant effects (*p* < 0.05) and non-significant effects (*p* > 0.05), respectively. The width of the paths indicates the level of significance, and *R*^2^ represents the proportion of the explained variance, Q^2^ represents the predictive relevance (model’s goodness of fit: 0.529).

Random forest modeling reveals microbial predictors of ecosystem carbon storage (Figure 9), and indicates that the diversity of microorganisms involved in the four types of soil carbon metabolism significantly affected soil carbon storage (*p* < 0.05). Among them, the *α* diversity indices (Shannon and Simpson indices) and *β* diversity index of carbon-fixing microorganisms were important predictors of carbon storage of grassland ecosystems. In terms of relative abundance at the genus level, *Methylococcus*, *Methylomonas*, *Methylocapsa*, and *Methylocystis* were the important taxa among methanotrophs for predicting carbon storage, while *Azospirillum*, *Methylibium*, Var*iovorax*, *Bradyrhizobium*, *Cupriavidus*, *Thiobacillus*, and *Mesorhizobium* were the important taxa among carbon-fixing microorganisms. Additionally, among methanogens, *Methanosarcina* and *Methanoregula* were the important groups influencing carbon storage, and *Amycolatopsis*, *Actinoplanes*, *Chitiniphilus*, *Cellulomonas*, and *Stenotrophomonas* were the important taxa among chitinase-producing microorganisms for predicting carbon storage.

Figure 9 showed that SWC explained 89.3% of the variation in carbon storage in the alpine grassland. The *R*^2^ values for ecosystem carbon storage, methanotrophs diversity, carbon-fixing microorganism diversity, methanogens diversity, and chitinase-producing microorganism diversity were 0.893, 0.571, 0.775, 0.212, and 0.545, respectively, indicating that soil and vegetation characteristics of the grasslands significantly influenced their carbon storage and carbon metabolism microorganism diversity. SWC affected ecosystem carbon storage (path coefficient = −0.923), mainly by changing vegetation characteristics (path coefficient = 0.884) and soil physicochemical properties (path coefficient = 0.867). SWC enhanced the diversity of methanotrophs (path coefficient = 0.721) and reduced the diversity of carbon-fixing microorganisms (path coefficient = −0.471) by increasing the biomass of vegetation. It also indirectly affected the diversity of methanotrophs by influencing soil physicochemical properties (path coefficient = −1.028).

## 4 Discussion

### 4.1 Effects of SWC on carbon storage in different alpine grassland ecosystems

The study found significant differences in carbon storage among the three types of alpine grassland ecosystems, with the relativity following the order of alpine wetland (AW) > alpine meadow (AM) > alpine desert (AD) (Figure 2b), an outcome consistent with previous findings (Li Z. et al., 2023; Wang et al., 2022). This study further revealed that surface SWC in the alpine grasslands is a key factor contributing to these differences in carbon storage (Figure 9). SWC influences vegetation carbon content by affecting vegetation indices (vegetation diversity and biomass) and also alters soil carbon storage in alpine grasslands by influencing soil physicochemical properties (Figure 9).

SWC directly affects vegetation carbon storage by altering plant community structure and biomass (Figure 9). A high SWC promotes the growth of sedges with a high carbon content and increases the availability of soil nutrients and moisture, thereby enhancing belowground vegetation biomass and resulting in the highest vegetation carbon storage in AW (Yuan et al., 2019; Zhang et al., 2011). In contrast, the alpine desert with the lowest SWC has a low vegetation biomass and a high proportion of forbs with a low carbon content (Table 2), leading to the lowest vegetation carbon storage (Yuan et al., 2019). Additionally, SWC has a direct and significantly positive effect on soil carbon storage (Figure 9), with high SWC environments being more conducive to soil carbon accumulation (He et al., 2012; Balasubramanian et al., 2020). Therefore, AW with the highest SWC has the highest vegetation and soil carbon storage, while AD with the lowest SWC has the lowest carbon storage.

### 4.2 Differences in soil physicochemical properties among different grassland types

SOM, NH_4_^+^-N, TN and TC in AW were significantly higher than those in AM and AD, while pH was significantly lower (*p* < 0.05) (Table 2). These findings are consistent with those in previous studies (Liu et al., 2019). This is mainly because increased SWC promotes plant growth and litter input, thereby increasing soil SOM and TC content (Jobbágy and Jackson, 2000). In addition, a high SWC also promotes the nitrogen mineralization process, thereby increasing soil NH_4_^+^-N content. It can also reduce nitrogen volatilization and leaching losses, leading to an increase in soil total nitrogen (TN) (Delgado-Baquerizo et al., 2013). Conversely, a higher soil moisture content enhances soil microbial activity, promoting the dissolution and migration of potassium ions, resulting in a decrease in TK content (Chen Y. S. et al., 2022). Additionally, increased SWC may lead to an increase in acidic substances in water (such as carbonic acid formed by the dissolution of carbon dioxide), thereby lowering soil pH (Qian et al., 2023).

### 4.3 Effects of SWC on the diversity and composition of soil carbon-metabolizing microorganisms

The PLS-SEM model indicated that SWC in different grasslands indirectly affects the diversity of methanotrophs and soil carbon-fixing microbes by influencing soil physicochemical properties (SOM, TK, NH_4_^+^-N, TN, TC, pH) and vegetation characteristics (BGB) (Figure 9).

There were significant differences in the *α*-diversity (richness, Shannon, Simpson indices) of carbon-metabolizing microorganisms among the three types of alpine grassland, with the lowest α-diversity for all four types of microorganisms (methanotrophs, carbon-fixing microbes, methanogens, and chitinase-producing microorganism) observed in AW (Figure 3). Additionally, the *β*-diversity of these microorganisms in AW also differed significantly from that in AM and AD (Figures 4e–h). SWC was a key factor contributing to these differences. The high SWC in AW led to poor soil aeration, significantly inhibiting the activity and diversity of aerobic or facultative anaerobic microorganisms (such as soil carbon-fixing microbes, methanotrophs, and chitinase-producing microorganism) (Delgado-Baquerizo et al., 2016; Tang et al., 2023; Li et al., 2024). Although SWC was positively correlated with the *mcrA* gene (Wei et al., 2018), the hypoxic environmental conditions in AW may have selectively favored them to become the dominant groups, thereby reducing the overall diversity of methanogens (Narrowe et al., 2017). Previous studies have also found that plant biomass can highly influence soil microbial activity (Hassan et al., 2019; Schultz et al., 2011). SWC significantly increased the diversity of methanotroph communities by increasing vegetation BGB but decreased the diversity of carbon-fixing microbial communities (Figure 9).

This study also found that the four carbon-metabolizing microorganisms had a similar community composition in all grasslands, with Pseudomonadota being the dominant phylum for soil methanotrophs and carbon-fixing microorganisms, Euryarchaeota being the dominant phylum for soil methanogens, and Pseudomonadota and Actinomycetota being the dominant phyla for chitinase-producing microorganisms (Figure 5). However, the relative abundances of key dominant phyla varied among the grasslands, due to the influence of SWC. Specifically, the relative abundance of Pseudomonadota, the dominant phylum for carbon-fixing microorganisms, followed the order of AW > AM > AD (Figure 5b), which was strongly positively correlated with SWC (LEfSe results in Figure 7c). The lowest SWC in AD not only directly limited water supply but also reduced NH_4_^+^-N content, belowground biomass, and vegetation cover (Figure 7c). These environmental conditions, together with poor soil nutrients and low vegetation biomass due to the low SWC, jointly inhibited the growth of Pseudomonadota (Rambia et al., 2024). Conversely, the extremely high SWC in AW directly created a hypoxic environment that was highly favorable for the growth and methane production of the dominant phylum Euryarchaeota for methanogens, resulting in its highest relative abundance in AW (Reitschuler et al., 2016). The low SWC in AD led to a low vegetation biomass (Table 2), reduced soil organic input, and poor nutrient availability, while Actinomycetota gained a competitive advantage in this resource-limited environment due to its ability to decompose recalcitrant carbon (Tang et al., 2023). Therefore, SWC not only directly regulates the anaerobic environment required by methanogens (Euryarchaeota) but also affects the relative abundance of dominant phyla in the four carbon-metabolizing microbial communities by influencing water availability, nutrient cycling, and vegetation conditions.

**Figure 10:**
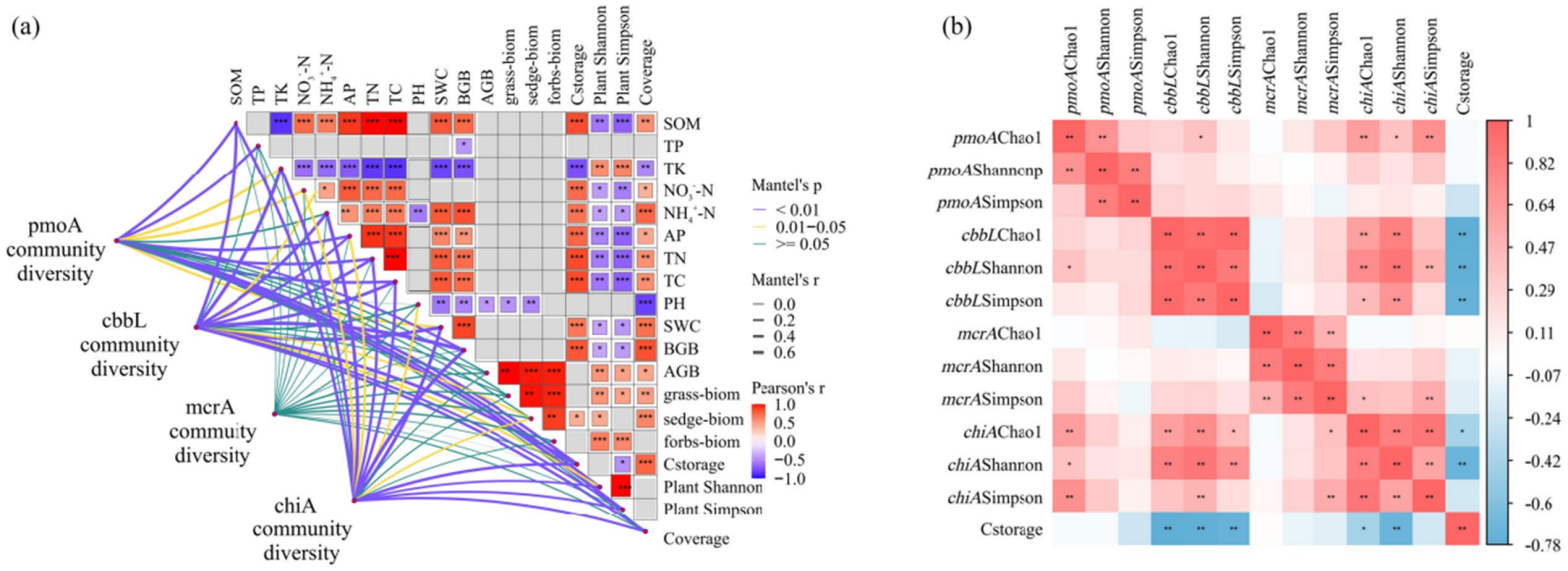
**(a)** Results of Mantel test showing the relationship between environmental factors and the community structure of soil methanotrophs, carbon-fixing microorganisms, methanogens, and chitinase-producing microorganisms; **(b)** Correlation coefficient between the diversity of methanotrophs, carbon-fixing microorganisms, methanogens, and chitinase-producing microorganisms and ecosystem carbon storage. * *p* < 0.05, ** *p* < 0.01, *** *p* < 0.001.

LDA analysis further revealed the effects of SWC on key functional microorganisms (Figure 10). The dominant methanotrophic genera *Methylosinus* and *Methylacidiphilum* in AW can only grow using methane as the sole carbon and energy source (Oshkin et al., 2020; Di et al., 2024), indicating that methane concentration produced by the hypoxic environment with a high SWC in AW was higher than that in AM and AD. The strict anaerobic carbon-fixing genus *Sulfuricaulis* and the methanogenic genus *Methanobrevibacter* dominated AW due to the strong selection for their growth and functions by the anaerobic conditions created by a high SWC (Spain et al., 2009; Kuo et al., 2024). The distribution patterns of these marker microorganisms demonstrated that SWC influences specific carbon-metabolizing functional microbial communities by regulating soil environmental conditions.

### 4.4 Relationships between soil carbon-metabolizing microorganisms and carbon storage

**Figure 8:**
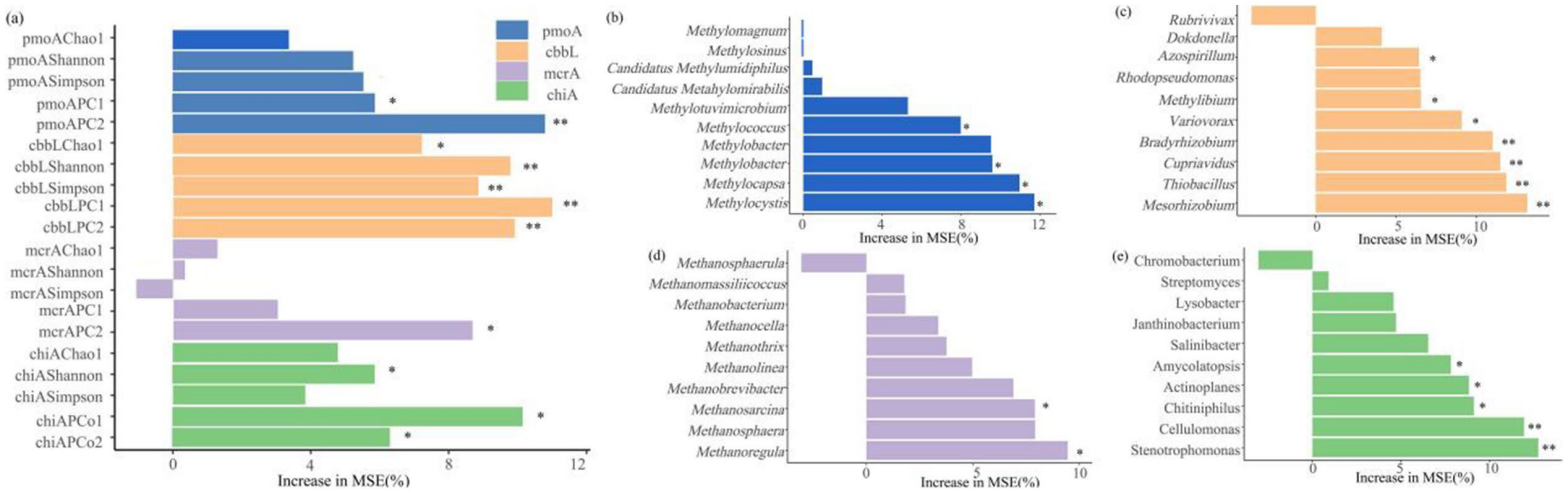
**(a)** Random forest modeled results of soil carbon storage based on the diversity of carbon metabolism microorganisms and the relative abundance of dominant genera among **(b)** methanotrophs, **(c)** carbon-fixing microorganisms, **(d)** methanogens, and **(e)** chitinase-producing microorganisms. The percent increase in mean squared error (MSE) of the variables was used to estimate the importance of the predictors, with higher MSE (%) values indicating greater importance. * *p* < 0.05, ** *p* < 0.01, *** *p* < 0.001.

The diversity and composition of soil carbon-metabolizing microorganisms affect carbon storage in alpine grasslands (Figure 8a). The *β*-diversity of methanotrophs significantly influenced carbon storage (*p* < 0.05) (Figure 8a), with key genera such as *Methylococcus* (Type I), *Methylomonas* (Type I), *Methylocapsa* (Type I), and *Methylocystis* (Type II) directly participating in methane oxidation (Liu et al., 2016; Li et al., 2022). The dominant *Methylocystis* (Type II) in AW had a higher methane oxidation efficiency, significantly enhancing the carbon fixation capacity of the system (Singh and Tate, 2007). The diversity of carbon-fixing microorganisms had an extremely significant effect on carbon storage (*p* < 0.01), but its *α*-diversity was negatively correlated with carbon storage (Figure 10b), possibly reflecting the inhibition of aerobic carbon-fixing microbial activity by the high SWC of AW. Key carbon-fixing microbial genera such as *Thiobacillus* (directly fixing CO₂), *Mesorhizobium* and *Azospirillum* (promoting plant carbon fixation), and *Methylibium* (oxidizing methane) influenced carbon accumulation through diverse pathways (Komaniecka et al., 2010; Estendorfer et al., 2020). The higher abundances of *Mesorhizobium*, *Azospirillum*, and *Methylibium* in AM than in AD explained why AM had a higher carbon storage than AD (Figure 6b). *Methanogenic* genera such as *Methanosarcina* and *Methanoregula* influenced carbon decomposition through methane production. The Shannon diversity of chitinase-producing microorganism significantly influenced carbon storage (Figure 8a), with key genera such as *Amycolatopsis*, *Actinoplanes*, *Chitiniphilus*, *Cellulomonas*, and *Stenotrophomonas* being important taxonomic groups for predicting carbon storage (Figure 8e). Among them, *Cellulomonas* and *Stenotrophomonas* had extremely significant effects on carbon storage (*p* < 0.01). These microorganisms can participate in the carbon cycle by degrading chitin, and an increase in their relative abundance may lead to an increase in carbon decomposition capacity in grasslands, thereby reducing carbon storage (Cai et al., 2023; Zhang et al., 2022). The dominance of *Stenotrophomonas*, *Chitiniphilus*, and *Amycolatopsis* in AD explained why it had the lowest carbon storage. Therefore, the abundance of specific functional microbial groups and their metabolic activities are the core microbiological mechanisms driving differences in carbon storage among different types of alpine grassland.

## 5 Conclusion

This study found that the carbon storage capacity of the three types of alpine grassland ecosystems follows the ranking order of AW > AM > AD. SWC can increase vegetation carbon storage and soil carbon storage by enhancing belowground biomass and total soil carbon content, ultimately affecting the carbon storage of grassland ecosystems. SWC enhances methanogen diversity and reduces soil carbon-fixing microbes diversity by promoting vegetation biomass, and it also influences soil carbon-fixing microbes diversity by affecting soil physicochemical properties. Random forest model revealed that the diversity of the four types of carbon metabolizing microorganisms can all influence grassland carbon storage, among which there is a significant negative correlation between the diversity of carbon-fixing microorganisms and grassland carbon storage. The carbon-fixing microorganisms *Thiobacillus*, *Mesorhizobium*, *Azospirillum*, and *Methylibium*, as well as the chitinase-producing microorganisms *Cellulomonas* and *Stenotrophomonas*, have a highly significant impact on carbon storage.

## Data Availability

The original contributions presented in the study are included in the article/Supplementary material, further inquiries can be directed to the corresponding author.
