# Supplementary material for: Ecosystem carbon storage and carbon metabolizing microorganisms in three types of grasslands on the Qinghai-Tibet Plateau
Source: Front Microbiol. 2025 Jul 25;16:1627840. doi: 10.3389/fmicb.2025.1627840 (PMC12332514; doi:10.3389/fmicb.2025.1627840)
Supplement: Supplementary file 1 [file Supplementary_file_1.docx]

**Supplementary Marterials**

Figure S1 Experimental Design


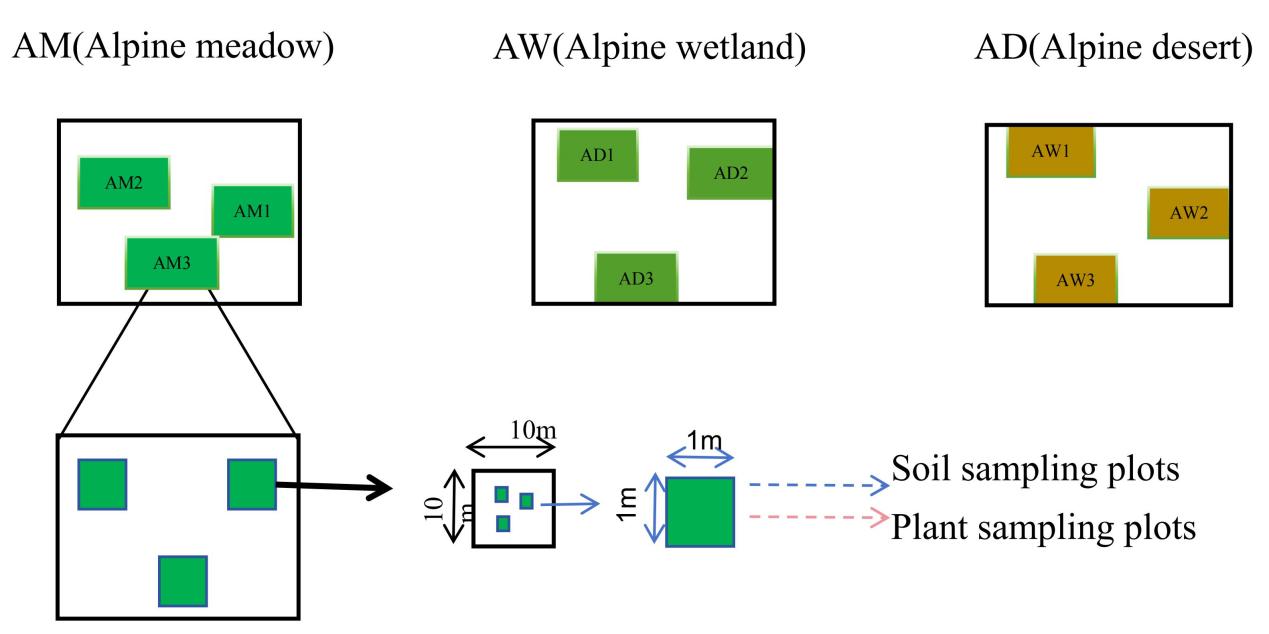


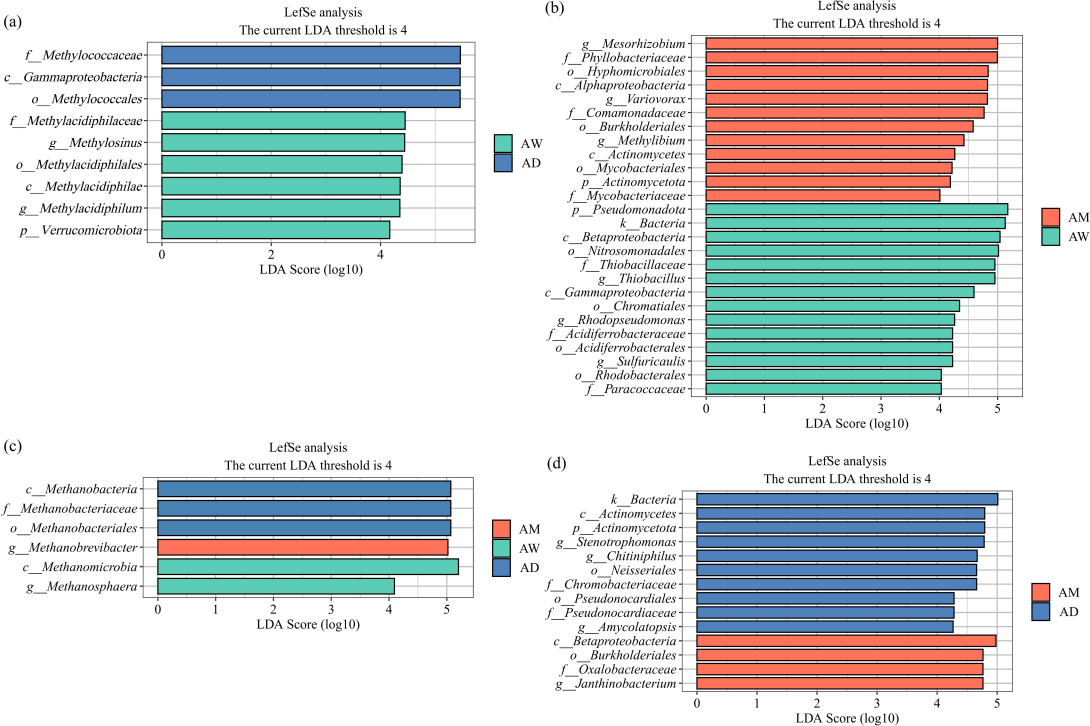


Figure S2. The histogram of LDA fraction of (a) methanotrophs, (b) carbon-fixing microorganism, (c) methanogens and (d) chitinase-producing microorganism community branches in soil of different types of alpine grassland was generated by LEfSe analysis, and the threshold value was 4.0. AM, alpine meadow; AW, alpine wetland; AD, alpine desert.

Table S1. Mantel test on the effects of environmental factors on the community structure, α-diversity index, and functional potential of methane-oxidizing bacteria, carbon-fixing microorganisms, methane-producing microorganisms, and chitinase-producing microorganisms.

| Type | Spec | env | r | P | env | r | p |
| --- | --- | --- | --- | --- | --- | --- | --- |
| *pmoA* | *pmoA* community | SOM | 0.411 | 0.004 | BGB | 0.257 | 0.005 |
|  |  | P | 0.362 | 0.001 | AGB | 0.077 | 0.110 |
|  |  | K | 0.273 | 0.010 | Grass−biom | 0.077 | 0.098 |
|  |  | NO_3_^-^-N | 0.319 | 0.016 | Sedge−biom | 0.058 | 0.132 |
|  |  | NH4+-N | 0.133 | 0.071 | Forb−biom | 0.111 | 0.072 |
|  |  | AP | 0.408 | 0.006 | Cstorage | 0.424 | 0.001 |
|  |  | TN | 0.423 | 0.003 | Shannon | 0.430 | 0.001 |
|  |  | TC | 0.424 | 0.003 | Simpson | 0.537 | 0.001 |
|  |  | PH | -0.073 | 0.806 | Coverage | 0.039 | 0.282 |
|  |  | SWC | 0.243 | 0.023 |  |  |  |
| *cbbL* | *cbbL* community | SOM | 0.478 | 0.001 | BGB | 0.731 | 0.001 |
|  |  | P | 0.029 | 0.341 | AGB | 0.025 | 0.245 |
|  |  | K | 0.585 | 0.001 | Grass−biom | 0.042 | 0.182 |
|  |  | NO_3_^-^-N | 0.216 | 0.043 | Sedge−biom | 0.132 | 0.03 |
|  |  | NH4+-N | 0.502 | 0.001 | Forb−biom | -0.002 | 0.395 |
|  |  | AP | 0.295 | 0.007 | Cstorage | 0.500 | 0.001 |
|  |  | TN | 0.444 | 0.001 | Shannon | 0.181 | 0.013 |
|  |  | TC | 0.487 | 0.001 | Simpson | 0.244 | 0.008 |
|  |  | PH | 0.145 | 0.051 | Coverage | 0.367 | 0.002 |
|  |  | SWC | 0.644 | 0.001 |  |  |  |
| *mcrA* | *mcrA* community | SOM | -0.045 | 0.668 | BGB | -0.041 | 0.765 |
|  |  | P | -0.033 | 0.647 | AGB | 0.101 | 0.053 |
|  |  | K | -0.028 | 0.604 | Grass−biom | 0.098 | 0.079 |
|  |  | NO_3_^-^-N | 0.001 | 0.434 | Sedge−biom | 0.085 | 0.083 |
|  |  | NH4+-N | -0.011 | 0.534 | Forb−biom | 0.104 | 0.061 |
|  |  | AP | -0.032 | 0.603 | Cstorage | -0.022 | 0.579 |
|  |  | TN | -0.032 | 0.598 | Shannon | -0.078 | 0.915 |
|  |  | TC | -0.040 | 0.639 | Simpson | -0.035 | 0.654 |
|  |  | PH | 0.026 | 0.333 | Coverage | 0.038 | 0.249 |
|  |  | SWC | -0.071 | 0.842 |  |  |  |
| *chiA* | *chiA* community | SOM | 0.391 | 0.001 | BGB | 0.634 | 0.001 |
|  |  | P | 0.022 | 0.375 | AGB | 0.013 | 0.325 |
|  |  | K | 0.448 | 0.001 | Grass−biom | 0.029 | 0.241 |
|  |  | NO_3_^-^-N | 0.207 | 0.021 | Sedge−biom | 0.107 | 0.049 |
|  |  | NH4+-N | 0.577 | 0.001 | Forb−biom | -0.002 | 0.414 |
|  |  | AP | 0.233 | 0.010 | Cstorage | 0.401 | 0.001 |
|  |  | TN | 0.353 | 0.002 | Shannon | 0.194 | 0.008 |
|  |  | TC | 0.389 | 0.001 | Simpson | 0.249 | 0.004 |
|  |  | PH | 0.174 | 0.021 | Coverage | 0.311 | 0.002 |
|  |  | SWC | 0.577 | 0.001 |  |  |  |
